# The CNS Stochastically Selects Motor Plan Utilizing Extrinsic and Intrinsic Representations

**DOI:** 10.1371/journal.pone.0024229

**Published:** 2011-09-02

**Authors:** Jindrich Kodl, Gowrishankar Ganesh, Etienne Burdet

**Affiliations:** 1 Department of Bioengineering, Imperial College of Science, Technology and Medicine, South Kensington Campus, London, United Kingdom; 2 Advanced Information and Communications Technology Research Institute, National Institute of Information and Communications Technology, Kobe, Japan; 3 Computational Neuroscience Laboratories, ATR International, Kyoto, Japan; Freie Universitaet Berlin, Germany

## Abstract

Traditionally motor studies have assumed that motor tasks are executed according to a single plan characterized by regular patterns, which corresponds to the minimum of a cost function in extrinsic or intrinsic coordinates. However, the novel via-point task examined in this paper shows distinct planning and execution stages in motion production and demonstrates that subjects randomly select from several available motor plans to perform a task. Examination of the effect of pre-training and via-point orientation on subject behavior reveals that the selection of a plan depends on previous movements and is affected by constraints both intrinsic and extrinsic of the body. These results provide new insights into the hierarchical structure of motion planning in humans, which can only be explained if the current models of motor control integrate an explicit plan selection stage.

## Introduction

To perform purposeful arm movements, e.g. moving objects and placing them in space, we need to activate muscles in order to fulfill task constraints. While the muscle system allows for infinite combinations of activations to execute a task [1], experiments have consistently shown that motor tasks are performed by using regular motion patterns (e.g. [2], [3], [4]). This suggests that the central nervous system (CNS) fulfills a process by which it distributes task dynamics among the muscles and the joints, although the exact coordinates in which planning occurs are still unclear. For example, [2] observed that arm movements involve straight line trajectories irrespective of the movement direction, and concluded that planning was in a space external to the body. However, these observations could be explained by minimization of a cost function in extrinsic [5] as well as in intrinsic coordinates [6], [7], [8], [9]. On the other hand, monkey electrophysiology studies have found neuronal activity related to variables in intrinsic [10], extrinsic [11] and multiple [12] coordinates, but it is unclear whether these observations represent parallel planning in multiple coordinates or merely exhibit coordinate transformations in the brain.

Furthermore, while the nature of the cost function has been debated over the years [13], [14], it has always been assumed that the CNS uses a unique motor plan to solve a given task, which may be generated through on-line optimal feedback control [8]. Multiple motor plans have only been studied in the context of multiple environments, e.g. dynamic environments [15], [16] or visuo-motor rotations [17]. On the other hand, while [18], [19], [20] have examined tasks with multiple solutions, it is unclear whether the observed patterns correspond to different plans or different movements, because no muscle invariant effects were observed.

What factors influence the development and selection of motor plans performed by the CNS? To examine this question systematically, we introduce a novel paradigm which can distinguish between the planning and execution phases of a movement from a behavioral perspective. We first selected a via-point task which was previously reported to exhibit multiple solutions [18]. Motivated by the motor memory identified in [20], we observed a similar effect in the via-point task, in the sense that ‘exploration’ of a particular solution influenced the selection of the subsequent solutions. In this paper we examine how subjects choose the trajectory in different orientations of the same via-point setup, and how the exploration of a particular solution affects the selection of a solution in unexplored orientations. This enables us to distinguish between a muscle invariant planning phase and a muscle dependent execution phase during movement. Our results show for the first time that the CNS selects from a multiple set of plans to perform the same task. The plan selection is influenced by constraints both intrinsic and extrinsic to the body.

## Results

**Figure 1:**
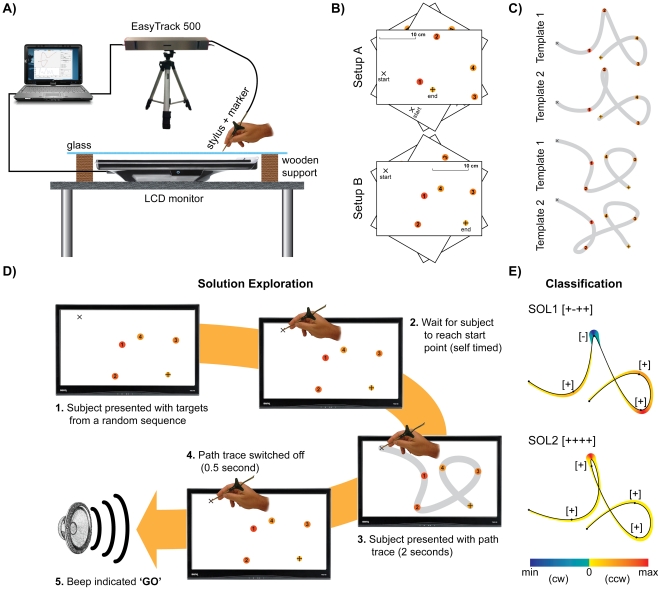
Experimental conditions. The setup (A) required subjects to make movements in various via-points configurations (B). Subjects were observed to choose predominantly two trajectories or solutions (C) which were utilized as templates during the exploratory phase (D). The two solutions were characterized by different curvature signatures as shown in (E).

Our experiment required subjects to hold a stylus and make movements in a horizontal plane (Fig. 1A) through 2 via-point *setups* (Fig. 1B) presented in three different orientations: 0°, 120° and 240° (see Methods for details). The six configurations (2 setups x 3 orientations) were presented pseudo-randomly in alternating *free sessions* and *trajectory exploration sessions*. For the same via-point setup we observed that subjects predominantly chose two different trajectories or *solutions: SOL1* and *SOL2* (Fig. 1C), which were classified by the curvature signature (Fig. 1E) and used as templates to guide the subject's movement in the exploratory sessions (cartoon of the sequence shown in Fig. 1D). Subjects performed only two orientations in the exploratory session (see Methods for details).

### Existence of multiple solutions

**Figure 2:**
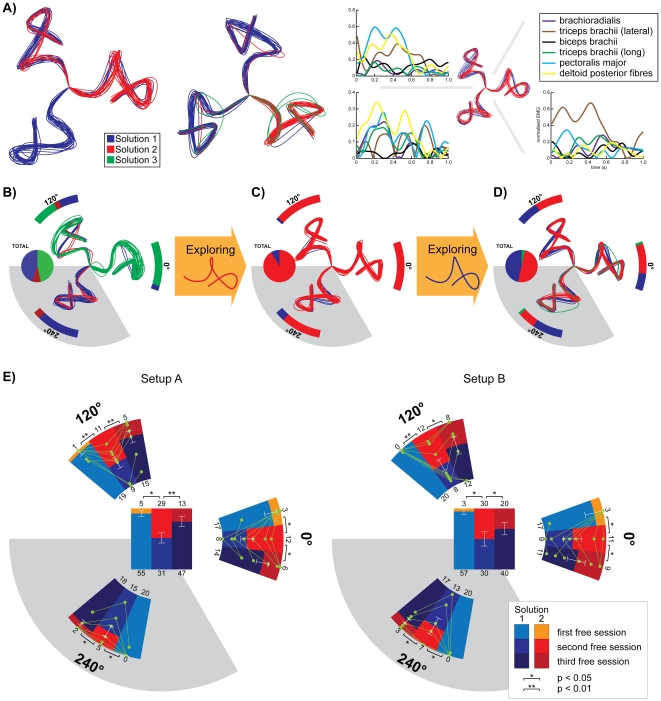
The via-point movements are spontaneously performed using multiple solutions. A) shows the trajectory solutions employed by two representative subjects in setups A and B, respectively. The trajectories in the three orientations have been separated out and placed in a radial arrangement for clarity. The difference in EMG patterns in six arm muscles between the two solutions is shown for a third subject in the individual orientations. The free trajectories of a third subject (B) show three different trajectories utilized in different proportions (pie chart) in the different orientations (arc bars). Following an exploration of SOL2 (C), the proportion of SOL2 increases dramatically in each orientation (arc bars) and in total (pie chart), showing an increase also in the unexplored orientation (grey). Subsequently, the free trials after exploration of SOL1 (D) show an increase of SOL1 in all orientations again. This effect was consistent across subjects (E) in both setups. The peripheral bar charts of (E) show the change in SOL1 and SOL2 across all subjects in the two explored and in the unexplored (grey) orientations. The central bar chart combines data from all orientations. Individual subject data is represented by the green traces.

Fig. 2A shows the hand paths of two representative subjects with the different orientations separated out. The subjects employed several solutions (different colors in Fig. 2A) to solve the task in each orientation. The different solutions (to the same setup) were characterized by paths with distinct curvature signatures (Fig. 2A), as well as different muscle activations (right panel of Fig. 2A). This confirms the observation of [18] that subjects vary between multiple patterns under different setup configurations, and gives us a tool to study motion planning.

**Figure 3:**
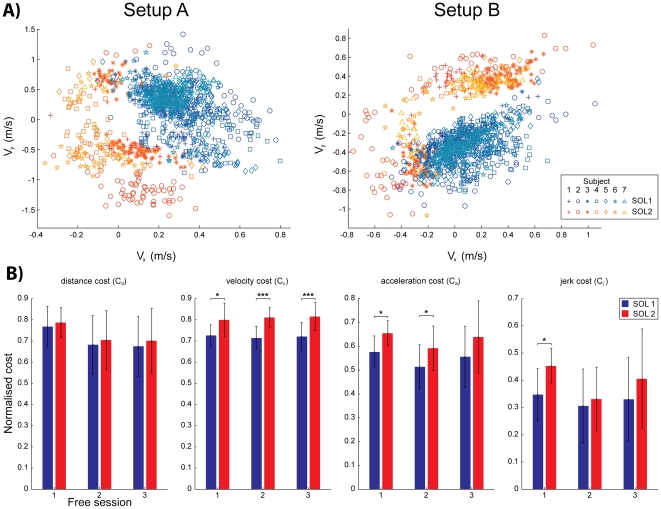
Quantifying the solution differences. A) To quantify the difference between the two subjects solutions, the *x* and *y* velocities of the hand were plotted from each trial of every subject (different markers) at their hand position closest to the second via point during execution of SOL1 (blue shades) and SOL2 (red shades). The velocity distribution was confirmed to be different (p<0.001, two-way ANOVA) for each subject. B) The execution costs calculated in terms of distance, velocity, acceleration and jerk were in mean systematically larger in SOL2 than in SOL1, however only the velocity cost was significantly higher. The error bars indicate standard error across subjects.

The choice of the solution was found to be random with no observable pattern in the selection. A few subjects used only one solution, and no subject used more than three solutions (see Table 1). To quantify the differences between the solution trajectories, we analyzed the velocities at the second via-point of setup A (first panel of Fig. 3A) and setup B (second panel of Fig. 3A) for SOL1 (blue) and SOL2 (red) and found them to be significantly different (p<0.001, two-way ANOVA) for each subject. SOL1 was kinematically optimal, in the sense that it had systematically less integral square velocity, acceleration and jerk than SOL2, though only velocity was significantly different (p<0.001, 2 sample T-test, Fig. 3B).

**Table 1:** 

		Setup A									Setup B								
		Session 1			Session 2			Session 3			Session 1			Session 2			Session 3		
		0°	120°	240°	0°	120°	240°	0°	120°	240°	0°	120°	240°	0°	120°	240°	0°	120°	240°
**Sub A**	**SOL1**	18	20	20	10	13	13	19	20	18	20	20	20	6	12	5	12	16	15
	**SOL2**	2	0	0	10	7	7	1	0	2	0	0	0	14	8	15	8	4	5
	other	0	0	0	0	0	0	0	0	0	0	0	0	0	0	0	0	0	0
**Sub B**	**SOL1**	19	20	20	3	0	20	7	3	20	20	20	20	2	3	20	7	0	19
	**SOL2**	1	0	0	17	20	0	13	17	0	0	0	0	18	17	0	13	20	1
	other	0	0	0	0	0	0	0	0	0	0	0	0	0	0	0	0	0	0
**Sub C**	**SOL1**	2	7	17	0	1	4	7	9	12	2	17	18	0	2	1	1	10	13
	**SOL2**	0	2	3	20	19	16	12	11	7	16	1	1	20	18	19	19	10	7
	**SOL3**	18	9	0	0	0	0	1	0	1	2	0	1	0	0	0	0	0	0
	other	0	2	0	0	0	0	0	0	0	0	2	0	0	0	0	0	0	0
**Sub D**	**SOL1**	7	15	20	4	10	19	16	15	20	20	20	20	19	17	20	19	19	19
	**SOL2**	13	5	0	16	10	1	4	5	0	0	0	0	1	3	0	1	0	1
	other	0	0	0	0	0	0	0	0	0	0	0	0	0	0	0	0	1	0
**Sub E**	**SOL1**	18	20	20	14	9	19	20	19	20	20	20	20	16	16	15	19	20	18
	**SOL2**	2	0	0	6	11	1	0	1	0	0	0	0	4	4	5	1	0	2
	other	0	0	0	0	0	0	0	0	0	0	0	0	0	0	0	0	0	0
**Sub F**	**SOL1**	20	20	20	7	12	10	11	17	18	20	20	20	10	5	12	16	15	16
	**SOL2**	0	0	0	13	8	10	9	3	2	0	0	0	10	15	8	4	5	4
	other	0	0	0	0	0	0	0	0	0	0	0	0	0	0	0	0	0	0
**Sub G**	**SOL1**	20	20	20	16	16	17	20	20	20	20	20	20	8	4	20	4	0	20
	**SOL2**	0	0	0	4	4	3	0	0	0	0	0	0	12	16	0	16	20	0
	other	0	0	0	0	0	0	0	0	0	0	0	0	0	0	0	0	0	0

Figs. 2B, 2C and 2D show the behavior of a third subject on exploration of a different solution, where the explored setup configurations were presented in 0° and 120° orientations. Initially in the free trials (Fig. 2B) the subject used three solutions in all the orientations. After exploration of SOL2, the choice of SOL2 increased in all orientations (see increase in red trace in Fig. 2C) including in the 240°-configuration which was not presented in the exploratory session. Subsequently, upon exploration of SOL1, the population of SOL1 increased (see increase of blue trace in Fig. 2D) in all orientations of movements. Again, an increase was observed even in the 240°-configuration, which had not been used in the exploratory session. These observations were consistent with every subject (see pattern of green traces in Fig. 2E and Table 1), in both of the setups and significant across subjects (see p-values in Fig. 2E). The behavior across subjects in each (peripheral plots) and across (central plots) orientations is shown in Fig. 2E.

**Figure 4:**
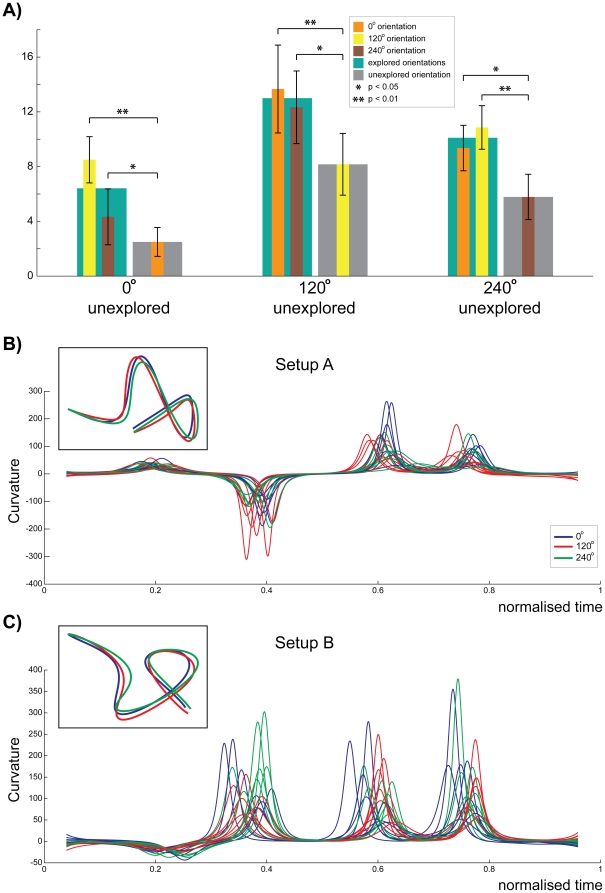
Orientation effect. (A) Explored orientations (turquoise bar) showed consistently larger effect than the unexplored orientation (grey bar), irrespective of the specific unexplored orientation. The mean trajectories of a representative subject during SOL1 in the different orientations (inset of B and C) and mean curvature of all the subjects (three color traces of B, C) show minor but significant (setup A: p_SOL1_<1e-16; setup B: p_SOL1_<1e-12) differences in the curvature profiles across orientations. Note that the curvature signature (and hence the solution classification) was maintained. Similar effects were also observed for SOL2 in both setups (setup A:p_SOL2_<1e-5; setup B:p_SOL2_<1e-16)

In the main and subsidiary experimental protocols, one of the three setup orientations was left unexplored in order to investigate the relationship between the change of solution proportion in the explored and unexplored orientations (Fig. 4A). Even though all orientations (both explored and unexplored) experienced an increase in the proportion of the previously explored solution, the increase observed in the unexplored orientation was significantly lower (p<0.0148) than for the explored orientations. This was valid individually for all the three experimental protocols, i.e. leaving 0° (p<0.01), 120° (p<0.01) or 240° (p<0.01) out of the exploratory session.

### Orientation-specific temporal and trajectory influence

Furthermore, among the same solutions orientation-specific deformations were observed in every trial (Fig. 4B,C) such that the curvature traces in the different orientations differed from each other both in setup A (p_SOL1_<1e-16 and p_SOL2_<1e-5) and setup B (p_SOL1_<1e-12 and p_SOL2_<1e-16) though the solution signature was maintained.

**Figure 5:**
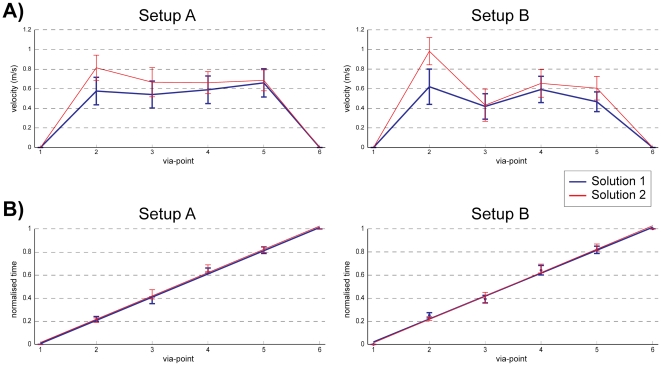
Velocity and time variation at via-points. The velocity plotted at the point of closest approach to the via-point for setup A and setup B (A), shows significant (p<0.001) differences between SOL1 (blue trace) and SOL2 (red). However, (B) the via-point passing time (normalized by the total movement time) is consistently similar regardless of setup or solutions (blue and red traces) with the time between each pair of consecutive via-points observed at one fifth of the total time (r = 0.996). All plots show across-subject means and standard error.

Velocity patterns changed with the different setups and solutions, e.g. the sum of differences of the velocity closest to the via-points were different in both setup A and setup B (p<0.001, Fig. 5A). However, the via-point passing time was found to be extremely consistent across all subjects, solutions, setups and orientations (Fig. 5B) (p>0.95).

### Subjects are not conscious of their planning strategy

The questionnaire completed by the subjects upon finishing the experiment indicated that they were unaware of the true number of unique configurations, with all subjects declaring significantly more setups than the two that were used. Furthermore, when asked if they perceived any relationship between the experienced configurations, more than half the subjects did not correctly identify the rotational change of the setups. Additionally, five of the seven subjects indicated that they did not employ any particular strategy, and the other two subjects described a strategy different from the one they actually used.

## Discussion

Most previous works on human arm movements (e.g. [5], [6], [8], [21]) assumed that the human CNS selects a single arm trajectory either prior to or during movement, which is executed by the muscle system. In sharp contrast, our results on via-point movements show that *subjects employed multiple hand trajectories as solutions for a same task* (Figs. 2A and 2B), switching randomly between these solutions. Questionnaire responses indicated subjects' naivety, i.e. the subjects were neither aware of the similarities between the target configurations, nor of the planning strategy used, which suggests that this selection corresponds to an unconscious mechanism.

The probability of using a specific solution was influenced by its previous exploration (Figs.2B–E). This result, in line with a recent study [20], helped us to behaviorally distinguish between motion planning and execution. The increases in the selection probability (Figs. 2E and 4A), even for movements in orientations requiring completely different muscle activations than the explored directions (different EMG pattern across orientations in Fig. 2A), reveal two points: 1) It exhibits the presence of a higher motor level invariant of the muscles which we refer to as *motor plan*; 2) It shows that the plan exists in the orientation invariant external space.

While previous studies [2], [23], [24] suggested such a motor plan by looking at regularities in the task space trajectories, they could not exclude the possibility that the regularity may still be due to intrinsic planning, as exhibited by Harris and Wolpert's model [7]. In contrast, in our paradigm, by looking at the trajectory transfer properties across muscle sets, we avoid intrinsic effects. Thus while our results agree with Morasso's hypothesis [2], we provide a stronger, independent proof of the existence of a motor plan.

Furthermore, we observed that the influence of exploration of a specific solution was consistently larger in the explored orientations in comparison to the non-explored one (Fig. 4A), indicating that *the orientation also influences the trajectory selection and hence planning*. This orientation factor may correspond to coding in intrinsic joint/muscle space coordinates as observed in [20], or in an orientation variant extrinsic space (such as visual space). Note that the effect of orientation on the selection of a motor plan is distinctly different from generalization effects observed with force field [22], where the endpoint force changes gradually (and trajectories change continuously) as one goes away from the orientation where the task was learnt. In our case, we have several distinct trajectories with distinct muscle activation patterns (as seen by curvature signatures) and with a change in orientation the probability to select a particular solution changes.

Finally, trajectories corresponding to the same solution exhibited minor kinematic differences (Figs. 4B, C), indicating possible local optimization in joint/muscle space [20], [21].

Therefore, it appears that *both extrinsic and intrinsic constraints influence the motor plan and execution in the via-point task*. While the selection is conditioned by the task definition in extrinsic and possibly also intrinsic spaces, it is reinforced by motion execution in intrinsic coordinates.

A surprising observation in our task was the extreme regularity of the times at which via-points were passed by all the subjects. While an “isochrony principle” has been reported in scribbling and tracing [25], [26], [27] in the form of similar timing independent on the size of a pattern, we found that in movements through multiple via-points, the time interval between passes of two consecutive via-points was consistently one–fifth (r = 0.996 for regression line) of the total movement time. This is true even though distance between the via-points is not similar (p<1e-5, one-way ANOVA) in both the setups and was irrespective of via-point arrangement, orientation, and even solution choice. Fig. 5 shows the variation of the via-point time in all the subjects with the variation observed for velocity. However, further experiments are required to analyze this aspect with larger differences of distance between consecutive via-points.

Which mechanism would be consistent with all results of this experiment? The presence of multiple solutions affected by previous execution cannot be directly explained by previous motor control models. The presence of a movement plan separated from the execution is not compatible with online optimal feedback control [8], which assumes that motion is developed online during execution. In contrast, our results show that movement is determined not only by the current execution, but also by the history of previous movements. In general, the presence of multiple solutions is not compatible with global optimization models [5], [28], [7], [8] or models assuming a desired reference trajectory [21], [29]. Namely, these models require to integrate an additional plan selection level to explain our results. On the other hand, the very regular via-points timing suggests that it may be a more important determinant of motion planning than kinematic variables such as velocity, acceleration or jerk. This regular timing may be explained if motion plan corresponds to the limit cycle of an oscillator [30], [31]. Trial by trial variability can explain why the other solutions are still selected in some trials, even though the parameters of the oscillator tend to repeat the explored trajectory. Finally, motion generation dynamics and local optimization (e.g. [21]) would explain systematic variations observed in the different directions.

In summary, we introduced a paradigm which clearly distinguished the different planning and execution phases of movement behaviorally. With this paradigm: 1) We showed that tasks can have multiple possible solutions, in which case the human CNS stochastically selects from the several possible plans; 2) We also provided a stronger, independent proof for the previous proposition that planning is done in coordinates extrinsic to the body; 3) Additionally, our results revealed for the first time that intrinsic constraints also influence motor planning; 4) Finally, we found that the current motor control models are required to integrate an explicit plan selection stage in order to explain these observations.

## Methods

### Subjects

13 naive right-handed subjects (2 females) without known pathology, aged between 21 and 30 years, participated in a main experiment (7 subjects) and in subsidiary experiments (6 subjects). The experiments were conducted according to the principles in the Declaration of Helsinki and were approved by the ethics committee at Imperial College London. Each subject gave an informed consent prior to involvement in the study.

### Experimental setup

Each subject sat in front of a glass-protected horizontally laid LCD monitor and was asked to hold a stylus in his right hand (Fig. 1A). The subject's seated position and height were adjusted so he or she was able to comfortably reach any position on the glass panel covering the monitor, allowing for a free motion within the active range. Additionally, it was ensured that the center of the body was aligned with the center of the monitor and that the subject held his or her body upright at all times. No physical bindings were used to allow for natural, free movement. The stylus motion was recorded at 40 Hz by EasyTrack 500 optical motion tracking device (Altracsys LLC, Switzerland) using an active marker attached to the stylus to locate the stylus' tip. A second active marker was also attached to the system and used for the calibration. The graphical user interface and the acquisition software were implemented in MathWorks MATLAB R2007a with the software libraries necessary for controlling the hardware provided by Altracsys.

### Task

Subjects were repeatedly presented with a series of numbered points over three *free* sessions separated by two *trajectory exploratory session*s. They were required to “make a smooth and continuous movement from the ‘start’ to the ‘end’ targets via the ‘1’, ‘2’, ‘3’, ‘4’ targets, as fast and as accurately as possible”. The entire experiment lasted approximately 60 minutes. The subjects were allowed short breaks of about one minute in between each experimental session. No feedback of performance was given to the subject at any stage of the experiment.

The *free sessions* consisted of presenting the subject with two distinct setups (setup A and B) of targets, with both setups appearing in three possible orientations (Fig. 1B), obtained by rotating the setups 0°, 120° or 240°, reaching the total of six different configurations. The subject performed 20 trials in each configuration, equaling 120 trials (2 setups × 3 orientations × 20 trials), which were presented in random order. When a point configuration was presented, the subject was asked to locate and position the stylus tip at the starting point. An audio cue (‘go’) was given 1-2 seconds after settling into the starting position and the subject was required to begin his movement immediately upon hearing the cue. On completion of his movement, the subject was asked to remain at the ‘end’ target until the next configuration replaced the previous, upon which the whole cycle repeated.

The signed curvature *k*  =  (*x*′*y*″ -*y*′*x*″)/(*x*′^2^+*y*′^2^)^3/2^ of each trajectory in the Cartesian coordinates (*x*,*y*) was computed, allowing for a classification of the trajectories by the sign of curvature nearest to the via-point as illustrated in Fig. 1E. The coordinates defined relative to the subject led to a positive curvature for anti-clockwise movements, and negative for clockwise movements. The resulting sets were labeled as Solution 1 (SOL1), Solution 2 (SOL2), Solution 3 (SOL3), etc., according to the level of appearance, with SOL1 being the most commonly used solution. Subsequently, SOL3 and higher were discarded if they accounted for <1% of all the responses. The curvature sign sequence of SOL1 and SOL2 for setup A shown in Fig. 1E are [+ - + +] and [+ + + +], respectively, and [- + + +] and [- - + +] for setup B, respectively.

In the *trajectory exploratory session* the subject was presented with only 0° and 120° rotations of the two setups opposed to the three rotations in the free session, with 30 trials in each configuration. All 120 trials (2 setups x 2 orientations x 30 trials) were again presented in random order. When a target configuration was presented, the subject located the start point similarly as in the free session. After settling into position, the subject was shown an alternate solution template, which was superimposed on the targets, but left the targets visible. The solution template was a 1.5cm wide semi-transparent strip of either of the solutions in Fig. 1C. The template for SOL2 was used during the first trajectory exploration session, while SOL1 was presented as the template in the second trajectory exploration session. The template remained visible for 2 seconds and the subject was asked to remember it. Next, the template was switched off, but the configuration targets remained visible for 0.5 seconds before the audio cue instructed them to repeat the memorized movement immediately (Fig. 1D). Upon completion of the movement, the subject was asked to remain on the ‘end’ target until new configuration replaced the previous one. The cycle was then repeated.

Once the subject had completed all 600 trials (120 trials × 3 free sessions + 120 trials × 2 trajectory exploration sessions) he or she was given a questionnaire with queries about the strategy and any changes to the strategy used during the experiment, as well as any perceived relationship between the presented configurations.

A subsidiary experiment was performed in order to examine whether the observed patterns were specific to the explored orientations. The experiment protocol and sessions in the subsidiary experiment were similar to those in the main experiment. The only difference was the orientations used in the exploration session. Three subjects were presented with 120° and 240° rotations and the remaining three with 0° and 240° rotations.

The subjects' responses were smoothened using a third order low-pass Butterworth filter with 5Hz cut-off frequency to eliminate the noise.

### Muscle activation

Surface electromyography (EMG) was recorded from a subject to exhibit the muscle activation differences between the various solutions and rotations. The major proportion of the movement whilst performing the task was identified around the elbow and shoulder joints. Electromyography (EMG) activity was recorded from six muscles that greatly contribute to the control of these joints; brachioradialis (forearm flexion), triceps brachii lateral head (elbow extension), biceps brachii (elbow flexion, forearm supination), triceps brachii long head (shoulder joint stabilization), pectoralis major (shoulder joint control) and posterior deltoid (shoulder flexion, abduction and extension). Each muscle was identified using the functional movements, and electrodes were attached accordingly.

The muscle activity investigation consisted of two forced sessions, where the subject was guided to perform SOL1 during the first session and SOL2 during the second session. In each session the subject was first shown 80 trials from the same sequence of setup configurations as during the first free session in the main experiment, ensuring that each configuration was performed at least 10 times. The corresponding solution templates were permanently superimposed on the configuration targets, with the targets still visible. When the configuration was presented to the subject, the subject was asked to locate the starting point.

Once in position, an audio ‘go’ cue prompted the subject to initiate the movement first by briefly (1 second) co-activating the muscles in his right arm and then beginning the movement as indicated by the visible trajectory template. After completing the motion, the subject was asked to remain at the ‘end’ target with his arm relaxed until the appearance of the next configuration.

The EMG signals were amplified using a g.tec commercial EMG amplifier (g.BSamp) and read into the computer using National Instruments data acquisition card (NI 6221). The read-in channels were filtered between 20 and 250 Hz, rectified and filtered using 5Hz cut-off frequency. The EMG signals of each setup and setup configuration was aligned to the start of the movement and averaged, obtaining 36 averaged EMG signals (2 setups x 3 configurations x 6 muscles) for both SOL1 and SOL2. Finally, each of the resulting 72 muscle signals was normalized by the mean activity of the corresponding muscle at the corresponding configuration.

### Statistics

To analyze the effect of the exploration sessions on the orientations, the choice number of SOL1 between free sessions (Fig 2E) were compared using 2 sample T-tests across the seven subjects.

Comparison of trajectories performed before and after exploration and across different orientations (Fig. 4) was performed using a two-way ANOVA between mean curvature trajectories of the seven subjects in the different sessions/orientations. A similar procedure was used to compare the peak velocity and time patterns of Fig. 5A.
